# Functional Study of Four Histone Genes Involved in the Spermatogenesis of *Cynoglossus semilaevis*

**DOI:** 10.3390/ani15040593

**Published:** 2025-02-18

**Authors:** Xuexue Sun, Zhijie Li, Lijun Wang, Haipeng Yan, Xihong Li, Na Wang, Zhongdian Dong, Wenteng Xu

**Affiliations:** 1 Guangdong Provincial Key Laboratory of Aquatic Animal Disease Control and Healthy Culture, College of Fisheries, Guangdong Ocean University, Zhanjiang 524088, China; s1506251002@163.com (X.S.); wnglikun@163.com (L.W.); yanhaipeng265438@gmail.com (H.Y.); 2 State Key Laboratory of Mariculture Biobreeding and Sustainable Goods, Laboratory for Marine Fisheries Science and Food Production Processes, Yellow Sea Fisheries Research Institute, Chinese Academy of Fishery Sciences, Qingdao Marine Science and Technology Center, Qingdao 266071, China; zhijieli2023@163.com (Z.L.); lixh@ysfri.ac.cn (X.L.); wangna@ysfri.ac.cn (N.W.)

**Keywords:** Chinese tongue sole, histone, spermatogenesis, histone modification, siRNA knockdown, mass spectral analysis

## Abstract

Chinese tongue sole (*Cynoglossus semilaevis*) is an important mariculture fish in China, and female individuals present a growth advantage. However, genetic females (ZW) can sex reverse to phenotypic males, designated pseudomales. The pseudomale shows abnormal spermatogenesis and produces only Z sperm. Histone is pivotal in spermatogenesis, and post-translational modification could regulate its function. A comparison of testis phosphorylated and ubiquitinated proteins revealed 8 and 12 differentially phosphorylated and ubiquitinated histones in the testes of male and pseudomale Chinese tongue soles, respectively, but there was no difference in the translation level of these proteins. We selected four histone genes, *h1.1-like*, *h1.2-like*, *h3*, and *h3.3-like*, for further analysis. The expression levels of the *h1.1-like*, *h3*, and *h3.3-like* genes reached their highest levels at 2 years post-hatching (yph), and the expression level of *h1.2-like* reached its highest level at 1.5 years post-hatching (1.5 yph), indicating that its role began during the late stage of gonadal development. Promoter activity verification revealed that the promoters of the *h1.1-like*, *h1.2-like*, *h3*, and *h3.3-like* genes were located approximately upstream 2000 bp and six histone-related transcription factor sites were predicted. YY1A, YY1B, C-JUN, and JUNB may have negative regulatory effects on *h1.1-like*, *h1.2-like*, *h3*, and *h3.3-like*; AR and ETS-2 may have positive regulatory effects on *h3* and *h3.3-like*. The ISH results revealed that *h1.1-like*, *h1.2-like*, *h3*, and *h3.3-like* mRNAs were located mainly in the sperm cells in the testes and the oocytes at various stages in the ovaries. After siRNA knockdown, the expression of dmrt1 in testis cell lines and the expression of *tesk1* and *neurl3* in males was downregulated, suggesting that the *h1.1-like*, *h1.2-like*, *h3*, and *h3.3-like* genes may have a negative regulatory role in spermatogenesis. The regulatory role in female fish remains to be explored. Mass spectrometry analysis revealed that histones have an important role in chromosome remodeling. These results provide a genetic basis for the molecular mechanism of gonadal development and spermatogenesis in Chinese tongue sole.

## Simple Summary

The genetic females of Chinese tongue sole (ZW sex chromosomes) can sex reverse to phenotypic males, designated pseudomales, while the pseudomale produces only Z sperm with W sperm missing. Previous studies have shown that histone plays an important role in spermatogenesis; thus, we selected four histone genes, *h1.1-like*, *h1.2-like*, *h3*, and *h3.3-like*, for further analysis. Their expression reached their highest levels at 1.5–2 years post-hatching, indicating that its role began during the late stage of gonadal development. The promoter of the four genes was located approximately 2000 bp upstream, and transcription factor sites were predicted. Among them, YY1A, YY1B, C-JUN, and JUNB may have negative regulatory effects on *h1.1-like*, *h1.2-like*, *h3*, and *h3.3-like*; AR and ETS-2 may have positive regulatory effects on *h3* and *h3.3-like*. In situ hybridization showed mRNAs of these four genes were located mainly in the germ cells in gonads. siRNA knockdown of the four histone genes could affect the genes related to spermatogenesis. These results provide a genetic basis for the molecular mechanism of gonadal development and spermatogenesis in Chinese tongue sole.

## 1. Introduction

Aquatic animals, especially many fish, exhibit sex growth dimorphism. Therefore, it is economically important to use sex control technology in important cultured fish and cultivate unisexual seedlings. The Chinese tongue sole is one of the nine major species of marine fish in East Asia. As one of the fish with the most significant difference in growth between males and females, the body weight of 1-year-old female fish can reach 2–4 times that of male fish [1]. The Chinese tongue sole has a unique phenomenon of spermatogenesis. The pseudomale fish (genotype ZW, but the phenotype is male) lack W-type sperm and can only produce Z-type sperm, whereas Z-type sperm carry paternal epigenetic information. The resulting offspring are more likely to become pseudomale fish [2,3,4]. For these reasons, the proportion of males can reach 80–90% of the whole population, which severely hinders the development of the industry [5].

Sex dimorphism and sex reversal make the Chinese tongue sole the preferred species for the study of sex determination, gonadal differentiation, and sex control technology [6]. Some researchers have attempted to achieve WW hypergamy by gynogenesis or the breeding of pseudomales and females and have finally found that pseudomales can produce only Z-type sperm [7]. In sexual reproduction, sperm, as a carrier of paternal information, is a key factor in species continuation. Spermatogenesis involves spermatogonia, primary and secondary spermatocytes, round spermatids, and mature spermatids. In the process of round spermatids forming mature spermatids, chromatin is remodeled, and the cell morphology changes significantly [8]. Histone-to-protamine transition during spermiogenesis is a critical part of this biological process. Histones are initially replaced by testis-specific histone variants, then transition proteins integrate into the nucleus and are in turn replaced by protamine [9]. Histones have five main families: H1/H5, H2A, H2B, H3, and H4 [10]. In comparison, histone variants are more likely to be modified by methylation, acetylation, phosphorylation, SUMOylation, and ubiquitination [11,12]. Histone variants can alter nucleosome and chromatin structure to regulate gene transcription. Histone variants are expressed not only in the S phase but also in other phases of the cell cycle, although their overall expression levels are relatively low. These histone variants possess unique biophysical properties; some can modulate nucleosome structure, while others can bind to specific regions of the genome [13].

However, it is a strange and interesting phenomenon in which Z-type sperm can be produced normally, whereas W-type sperm disappears’, and the reason remains ambiguous [14]. Early transcriptomic, phosphoproteomic, and ubiquitin proteomic analysis of the gonads of the Chinese tongue sole revealed that the regulatory factors that cause differences in gonadal differentiation and development between males and pseudomales were associated with histone modification. In the early stage, a series of genes were screened in the testes of male and pseudomale fish. There were differences in transcripts, but the differences in protein sequences were very small. A comparison of testis phosphorylated and ubiquitinated proteins revealed 8 and 12 differentially phosphorylated and ubiquitinated histones in the testes of male and pseudomale Chinese tongue soles, respectively, but there was no difference in the translation level of these proteins [15]. Phosphorylation, as a classic histone modification, has been widely reported in spermatogenesis. Phosphoproteomics technology has been used to determine the potential mechanisms of male infertility or reproductive defects. For example, in the Chinese mitten crab, H4 histone phosphorylation was found to be closely related to the reproductive ability of male crabs, which can be used as an epigenetic marker for sperm maturity [16]. Ubiquitination plays an important role in fish sex differentiation and spermatogenesis. For example, Ubiquitin carboxyl-terminal hydrolase L1(UCH-L1), a deubiquitinating enzyme, is highly expressed in the gonadal transformation and gametogenesis processes of eel and may play an important regulatory role [17]. More than 400 genes associated with spermatogenesis, including several ubiquitination-related genes, have been identified in Senegalese sole [18]. Research on testicular sperm phosphorylation and ubiquitination has not only helped to elucidate the biological basis of gonadal differentiation but also provided new perspectives for the identification of biomarkers for the regulation of sex control in aquatic fish.

We selected four histone genes, *h1.1-like*, *h1.2-like*, *h3*, and *h3.3-like*, with both ubiquitination and phosphorylation modifications for analysis and research. These data suggest its role in sex differentiation and spermatogenesis and provide new resources for exploring the mechanism of abnormal spermatogenesis and the development of sex control technology in fish.

## 2. Materials and Methods

### 2.1. Ethics Statement

The animal experiment was inspected and approved by the Institutional Animal Care and Use Committee at the Yellow Sea Fisheries Research Institute, CAFS (Approve No.: YSFRI-2022035).

### 2.2. Sample Preparation

Chinese tongue sole was obtained from the Haiyang Aquaculture Experimental Base (Haiyang, China). Tail fins were cut from each fish for genetic sex identification. PCR was performed by the primers Sex-F and Sex-R (Table S1), and the products were subjected to gel electrophoresis at 4% agarose concentration and 150 V voltage for 25 min [19]. In this study, heart, spleen, intestine, skin, muscle, gill, kidney, brain, liver, and gonad samples were collected from 8-month-old female and male Chinese tongue soles, quickly frozen in liquid nitrogen, and stored at −80 °C. The gonads of Chinese tongue soles at different developmental stages were also collected, including 40 days, 60 days, 90 days, 6 months, 7 months, 1 year, 1.5 years, and 2 years after hatching (40 dph, 60 dph, 90 dph, 6 mph, 7 mph, 1.5 yph, and 2 yph). Each stage included three males and females. The incubation conditions that induce spawning are a water temperature of 20.4–21.6 °C, a salinity of 35%, and pH of 7.8–8.6. The gonad samples were stored at −80 °C until RNA extraction. Simultaneously, the one-year-old fish (weight 97.97 ± 4.47 g, body length 24.25 ± 1.75 cm, body width 6.56 ± 0.96 cm) gonad samples were stored in 4% paraformaldehyde fixative and made into paraffin sections, and the wax blocks were sliced with 6 um thickness for in situ hybridization (ISH).

### 2.3. Gene Sequence Analysis and Alignment

Following the sequence (Gene ID = 103395672/103379258/103379247/103394976) of NCBI (https://www.ncbi.nlm.nih.gov) (accessed on 16 October 2024), the primers *h1.1-like*-F/R, *h1.2-like*-F/R, *h3*-F/R, and *h3.3-like*-F/R (Table S1) were designed to clone and verify the *h1.1-like*, *h1.2-like*, *h3*, and *h3.3-like* genes of Chinese tongue sole. ORF Finder (https://www.ncbi.nlm.nih.gov/orffinder/) (accessed on 20 October 2024) was used to infer the open reading frame and amino acid sequence. Expasy ProtParam (http://web.Expasy.org/protparam/) (accessed on 22 October 2024) and Expasy ProtScale (https://web.Expasy.org/protscale/) (accessed on 22 October 2024) were used to analyze the amino acid composition, molecular weight and theoretical isoelectric point of the *h1.1-like*, *h1.2-like*, *h3*, and *h3.3-like* proteins. TMHMM Server 2.0 (http://www.cbs.dtu.dk/services/TMHMM-2.0/) (accessed on 26 October 2024) was used to analyze the transmembrane region of the protein sequence. NetNGlyc1.0 (http://www.cbs.dtu.dk/services/NetNGlyc/) (accessed on 28 October 2024) and NetPhos3.1 (http://www.cbs.dtu.dk/services/NetPhos/) (accessed on 28 October 2024) predicted the protein glycosylation and phosphorylation sites of *h1.1-like*, *h1.2-like*, *h3*, and *h3.3-like* encoded amino acids. SignalP 6.0 (http://www.cbs.dtu.dk/services/SignalP/) (accessed on 29 October 2024) was used to analyze the potential signal peptide cleavage sites. Multiple sequence alignment software programs such as DNAMAN and MEGA 6.0 were used to cluster the amino acid sequences of multiple species and construct phylogenetic trees by the neighbor-joining method. The eighty-one species were used to construct the phylogenetic tree in this study. The partial species are as follows in Table 1.

### 2.4. Analysis of Gene Expression Patterns

RNA was extracted from different tissues by the RNA extraction kit (TianGen Biotech, Beijing, China). cDNA was synthesized by the PrimeScript^TM^ RT Kit (TaKaRa, Kyoto, Japan), and the final sample was stored at −20 °C. The primers *h1.1-like*-qF/R, *h1.2-like*-qF/R, *h3*-qF/R, and *h3.3-like*-qF/R (Table S1) were designed to analyze the expression patterns of the genes in different tissues and developmental stages. THUNDERBIRD^TM^Next SYBR^®^qPCR Mix (TOYOBO, Osaka, Japan) and the real-time fluorescent quantitative PCR system (Applied Biosystems, Waltham, MA, USA) were used for qPCR. The following protocol was set for qPCR: 95 °C for 30 s, 95 °C for 5 s, 60 °C for 30 s. Then, 40 cycles were performed. β-actin was used as the internal reference gene, and each group was repeated 3 times. The experimental data were calculated by the 2^−∆∆ct^ method [20]. SPSS 26.0 was used for multiple comparative analyses of the data by One-way ANOVA, and a *p*-value less than 0.05 was considered to indicate a significant difference between the two groups. In multiple comparisons, a and b are groups with different degrees of differential expression.

### 2.5. Cloning, Identification, and Activity Detection of the h1.1-like, h1.2-like, h3 and h3.3-like Gene Promoters

The primers *h1.1-like*-dF/R, *h1.2-like*-dF/R, *h3*-dF/R, and *h3.3-like*-dF/R (Table S1) were used to amplify the *h1.1-like*, *h1.2-like*, *h3*, and *h3.3-like* promoter regions. The pGL3-basic vector (Promega, Madison, WI, USA) was digested with the restriction enzyme HindIII for single enzyme digestion. The TOROIVD^®^ One Step Fusion Cloning Mix seamless cloning kit (TOROIVD, Baoshan, China) was used to insert the promoter fragment into the cut pGL3-basic vector to obtain pGL3-*h1.1-like*, pGL3-*h1.2-like*, pGL3-*h3,* and pGL3-*h3.3-like*. After the sequencing results were successfully compared with the NCBI sequence, the plasmid was extracted with the endotoxin-free small and medium extraction kit (TIANGEN Biotech, China), pGL3-control was the positive control, and pGL3-basic was the negative control. The experimental cells are derived from female human embryonic kidney cells and need to be cultured in DMEM (high glucose, containing L-glutamine, phenol red, sodium pyruvate, and free of HEPES, from Solarbio, Beijing, China) supplemented with 2% penicillin–streptomycin–amphotericin B mixed solution (Solarbio) and 10% fetal bovine serum (FBS). Moreover, they should be cultured at 37 °C and under 5% CO_2_ conditions. HEK 293T cells were transfected with pGL3-*h1.1-like*, pGL3-*h1.2-like*, pGL3-*h3*, pGL3-*h3.3-like*, pGL3-control, and pGL3-basic plasmids in 24-well plates by Lipo8000^TM^ transfection reagent (Beyotime, Nantong, China), and 500 ng of plasmid was added to each well. The luciferase activity of these cells was measured after 48 h by the dual luciferase reporter gene assay kit (Beyotime, China). The possible transcription factors of the *h1.1-like*, *h1.2-like*, *h3-like*, and *h3.3-like* promoters were predicted and screened by the online tool JASPAR (http://jaspar.genereg.net/) (accessed on 22 November 2024).

### 2.6. The Results of h1.1-like, h1.2-like, h3, and h3.3-like In Situ Hybridization (ISH)

The following primers with T7 polymerase and SP6 endonuclease sites were designed: *h1.1-like*-tF/R, *h1.2-like*-tF/R, *h3*-tF/R, and *h3.3-like*-tF/R (Table S1). Fragments of 448 bp, 441 bp, 352 bp, and 354 bp were amplified, and the PCR products were recovered. Following the kit (Roche in vitro transcription) and digoxin probe instructions, T7 or SP6 RNA polymerase was used for in vitro transcription to generate digoxin (DIG)-labeled antisense or sense RNA probes. Afterward, the probe was purified. The gonad samples of 1-year-old female, male, and pseudomale fish were sliced and fixed after dewaxing and hydration. Sections were pre-hybridized for 4 h at 70 °C. The pre-hybridization solution includes deionized formamide 25 mL + 20 × SSC 12.5 mL + H_2_O 15 mL + citric acid 460 μL + Tween 20 50 μL (tRNA final concentration 500 μg/mL and heparin final concentration 50 μg/mL). In identical liquid, an overnight incubation at 70 °Cwith probes (final concentration 0.2 g/mL) was performed on gonadal slices. Incubation with anti-DIG antibodies (Roche) was performed overnight after slices were blocked for 4 h at room temperature. A BCIP/NBT kit (Roche, Manheim, Germany) was used to generate the signal. A Nikon EClIPSE 80i microscope and a Pannoramic MIDI II (Hungary) were used to take photos.

### 2.7. SIRNA Interference (RNAi) of h1.1-like, h1.2-like, h3, and h3.3-like In Vitro and In Vivo and Its Effects on Related Genes

One siRNA targeting different sites of *h1.1-like*, *h1.2-like*, *h3*, *h3.3-like*, and the negative control (NC) was synthesized by Sangon (Shanghai, China) (Table S1). The Chinese tongue sole gonad cell lines were established in the laboratory by isolating the gonads. The established testicular and ovarian cell lines (derived from testes and ovaries and composed mainly of somatic cells) used for RNAi were performed [21]. The cells were cultured in an incubator at 24 °C and lived in the L-15 medium containing 2% penicillin–streptomycin–amphotericin B mixed solution (Solarbio), 5 ng/mL LIF (embryonic cell culture grade), 5 ng/mL BFGF, 5 ng/mL LIF, 14 μL mercaptoethanol and 15–20% fetal bovine serum (FBS). When the density reached 80–90%, the cells were covered with 12-well cell culture plates. RNA extraction and qPCR analysis were performed according to the above methods to measure the expression levels of the *h1.1-like*, *h1.2-like*, *h3*, and *h3.3-like* genes in cells. The genes siRNA sites were injected into 50-day-old Chinese tongue sole. These Chinese tongue soles were equally divided into a negative control group (NC) and an experimental group (*n* = 3 in each group). At 48 h after injection, the gonads were collected for gene expression analysis of *h1.1-like*, *h1.2-like*, *h3*, *h3.3-like*, and other sex-related genes (including foxl2, figla, dmrt1, cyp19a, sox-9, tesk1 and neurl3). The method was as described above, and the primers used in the experiment are shown in Table S1.

### 2.8. Histone Extraction and Mass Spectrometry Analysis

Histones were extracted with a histone extraction kit (Solarbio, China). One hundred milligrams of each tissue sample was cut into small pieces, and protein extract A was added. The tissue homogenate was homogenized to a nonobvious visible solid (2 μL of protease inhibitor mixture was added to each 500 μL of protein extract A and mixed and placed on ice for use). The tissue homogenate was transferred to a precooled, clean centrifuge tube and gently oscillated at 4 °C for 10 min. The mixture was subsequently centrifuged at 16,000× *g* for 15 min at 4 °C, the supernatant was discarded, and the precipitate was retained. One hundred microliters of histone extract B was added to the precipitate. The mixture was repeatedly mixed with 200 μL of the tip of the gun or fully vortexed to mix and precipitate, after which the mixture was placed in a refrigerator at 4 °C overnight and centrifuged at 16,000× *g* for 10 min, after which the supernatant was collected. The histone mixture was prepared by adding 25 μL of reagent C to the supernatant. Coomassie brilliant blue staining was performed after the gel was removed from the histone mixture, and the strip was cut and sent to Oebiotech (Shanghai, China) for mass spectrometry analysis.

## 3. Results

### 3.1. Bioinformatics and Phylogenetic Tree Analysis of h1.1-like, h1.2-like, h3, and h3.3-like Genes in Chinese Tongue Sole

According to Table 2, the *h1.1-like* is a hydrophilic stable protein (Figure S1). The *h1.1-like* protein has no transmembrane region or signal peptide cleavage site (Figure S2) and contains 26 phosphorylation sites, including 17 serine phosphorylation sites and 9 threonine phosphorylation sites, as well as 1 N-glycosylation site (Figure S3). According to the conserved domain prediction, the protein is H15 type (aa 31-96) (Figure S4).

According to Table 2, the *h1.2-like* is a hydrophilic unstable protein (Figure S1). The *h1.2-like* protein has no transmembrane region or signal peptide cleavage site (Figure S2) and contains 23 phosphorylation sites, including 13 serine phosphorylation sites, 9 threonine phosphorylation sites, and 1 tyrosine phosphorylation site, and it contains 0 N-glycosylation sites (Figure S3). According to the conserved domain prediction, the protein is H15 type (aa 28-93) (Figure S4).

According to Table 2, the *h3* is a hydrophilic unstable protein (Figure S1). The *h3* protein has no transmembrane region or signal peptide cleavage site (Figure S2); contains 19 phosphorylation sites, including 6 serine phosphorylation sites, 10 threonine phosphorylation sites, and 3 tyrosine phosphorylation sites; and contains 0 N-glycosylation sites (Figure S3). According to the conserved domain prediction, the protein is H3 type (aa 34-136) (Figure S4).

According to Table 2, the *h3.3-like* is a hydrophilic unstable protein (Figure S1). The *h3.3-like* protein has no transmembrane region or signal peptide cleavage site (Figure S2); it contains 19 phosphorylation sites, including 6 serine phosphorylation sites, 10 threonine phosphorylation sites, and 3 tyrosine phosphorylation sites, and it contains 0 N-glycosylation sites (Figure S3). According to the conserved domain prediction, the protein is H3 type (aa 34-136) (Figure S4).

**Figure 1:**
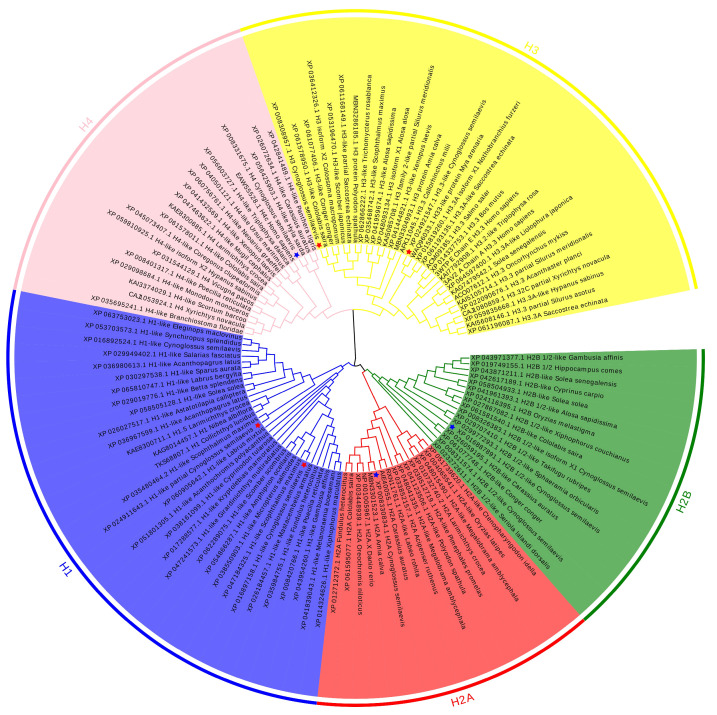
The *h1.1-like*, *h1.2-like*, *h3*, and *h3.3-like* sequences of multiple species constitute a phylogenetic tree, and the Chinese tongue sole is highlighted in red stars to show its evolutionary position.

Phylogenetic analysis revealed that the *h1.1-like* and *h1.2-like* genes of the Chinese tongue sole cluster and that the *h3* and *h3.3-like* genes also cluster, indicating closer kinship (Figure 1).

### 3.2. Expression Patterns of the h1.1-like, h1.2-like, h3, and h3.3-like Genes in Different Tissues and Sexes of Chinese Tongue Sole

**Figure 2:**
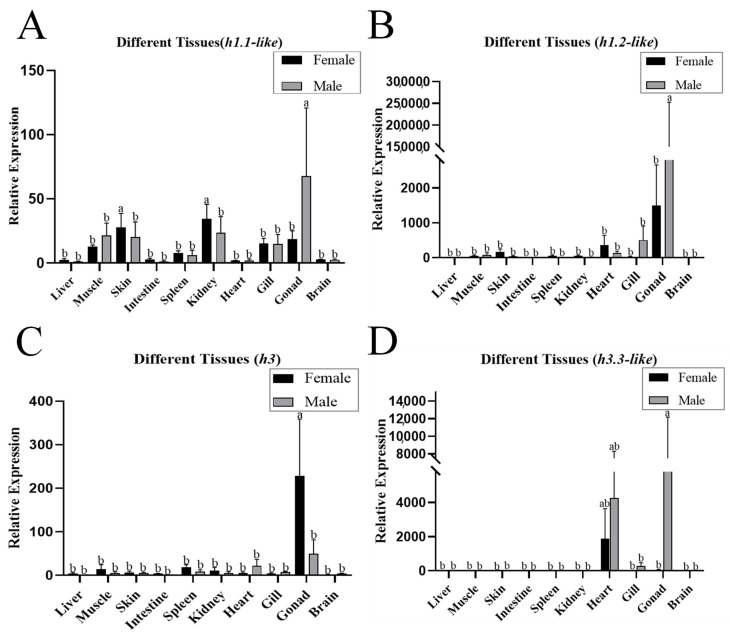
(**A**) Expression patterns of *h1.1-like* in different tissues. (**B**) The expression pattern of *h1.2-like* in different tissues. (**C**) Expression patterns of *h3* in different tissues. (**D**) Expression pattern of *h3.3-like* in different tissues. A *p*-value less than 0.05 was considered to indicate a significant difference between the two groups. In multiple comparisons, different alphabets indicated significant difference.

**Figure 3:**
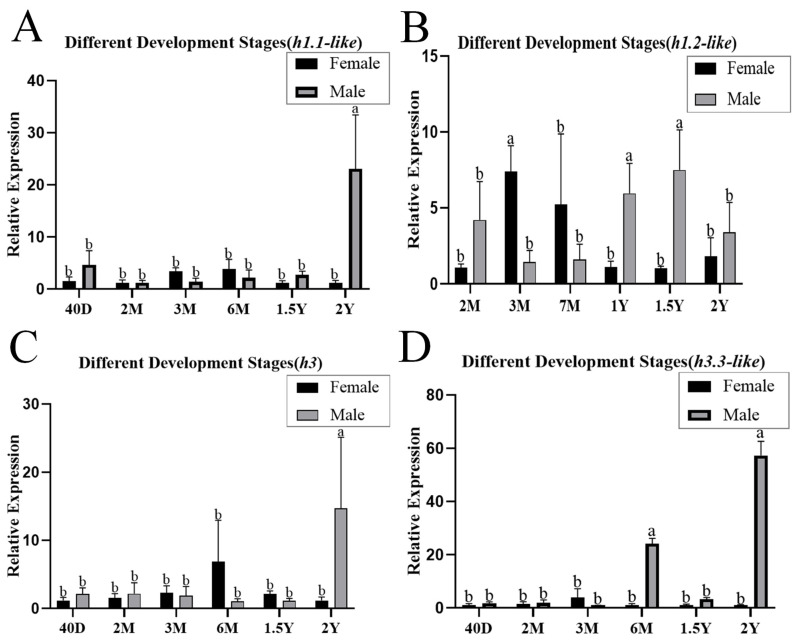
(**A**) Expression pattern of *h1.1-like* in different periods of gonadal development. (**B**) Expression pattern of *h1.2-like* in different periods of gonadal development. (**C**) Expression pattern of *h3* in different periods of gonadal development. (**D**) Expression pattern of *h3.3-like* in different periods of gonadal development. A *p*-value less than 0.05 was considered to indicate a significant difference between the two groups. In multiple comparisons, different alphabets indicated significant difference.

qPCR analysis revealed that *h1.1-like*, *h1.2-like*, *h3*, and *h3.3-like* mRNAs were widely distributed in all the detected tissues of females and males, with the highest expression in gonads and significantly greater expression in males than in females in the *h1.1-like*, *h1.2-like*, and *h3.3-like* genes (Figure 2). The *h1.1-like*, *h1.2-like*, *h3*, and *h3.3-like* genes were expressed at all developmental stages. In the testis, the expression levels of *h1.1-like*, *h3*, and *h3.3-like* were highest at 2 yph, and the expression level of *h1.2-like* was highest at 1.5 yph (Figure 3).

### 3.3. Detection and Analysis of h1.1-like, h1.2-like, h3, and h3.3-like Promoter Activity in Chinese Tongue Sole

**Figure 4:**
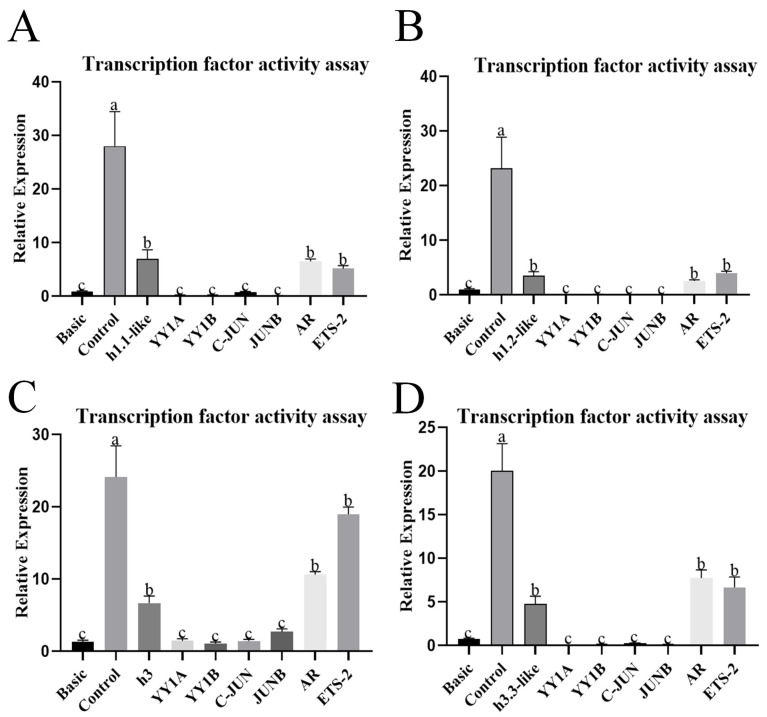
(**A**) Activity assay of transcription factor-binding sites on the *h1.1-like* promoter. (**B**) Activity assay of transcription factor-binding sites on the *h1.2-like* promoter. (**C**) Activity assay of transcription factor-binding sites on the *h3* promoter. (**D**) Activity assay of transcription factor-binding sites on the *h3.3-like* promoter. In multiple comparisons, different alphabets indicated significant difference.

Four promoters of 1928 bp, 1675 bp, 1940 bp, and 1974 bp were cloned in the upstream region of *h1.1-like*, *h1.2-like*, *h3*, and *h3.3-like* 5′UTR, which were subsequently transfected into HEK 293T cells and detected by dual luciferase activity. The *h1.1-like*, *h1.2-like*, *h3*, and *h3.3-like* promoters presented transcriptional activity. The promoter activity of the *h1.1-like*, *h1.2-like*, *h3*, and *h3.3-like* promoter regions decreased after binding to the four transcription factors YY1A, YY1B, C-JUN, and JUNB, whereas the promoter activity of the *h3* and *h3.3-like* promoter regions increased after binding to the AR and ETS-2 transcription factors (Figure 4).

### 3.4. Localization of h1.1-like, h1.2-like, h3, and h3.3-like mRNAs in the Gonads of Chinese Tongue Sole

**Figure 5:**
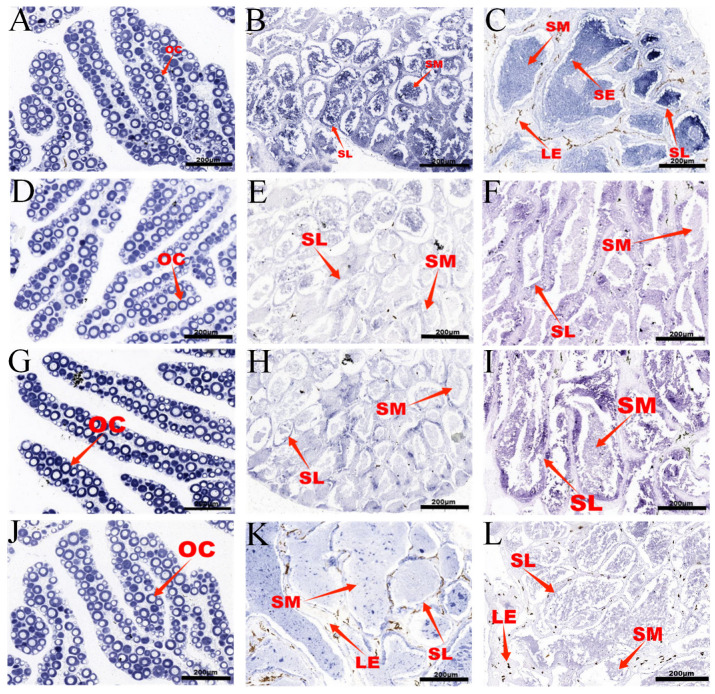
(**A**–**L**) Antisense probe (**A**–**C**) *h1.1-like* ISH results. (**D**–**F**) *h1.2-like* ISH results. (**G**–**I**) *h3* ISH results. (**J**–**L**) *h3.3-like* ISH results. (**A**,**D**,**G**,**J**) The antisense probe binds to the female. (**B**,**E**,**H**,**K**) The antisense probe binds to the male. (**C**,**F**,**I**,**L**) The antisense probe binds to the pseudomale. The fish fillets of each sample were 1 year old. SM: sperm, SL: lobule, LE: Leydig cells, SE: Sertoli cells, and OC: oocyte.

Primers with T7 polymerase and SP6 endonuclease sites were designed. T7 or SP6 RNA polymerase was used for in vitro transcription to generate digoxin (DIG)-labeled antisense or sense RNA probes, and slice in situ hybridization was performed. All section photos were obtained at 100× and 200× power (Figures S5–S8). The results revealed that *h1.1-like*, *h1.2-like*, *h3*, and *h3.3-like* genes were expressed mainly in the sperm cells in the testes and the oocytes at various stages in the ovaries (Figure 5).

### 3.5. RNAi-Mediated Knockdown of h1.1-like, h1.2-like, h3, and h3.3-like In Vitro and In Vivo and Its Effect on Sex-Related Gene Expression

**Figure 6:**
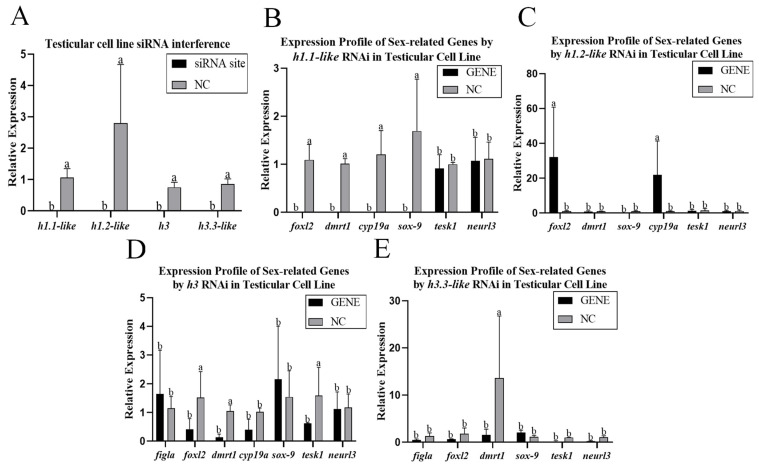
(**A**) Relative expression levels of *h1.1-like*, *h1.2-like*, *h3*, and *h3.3-like* in the testicular cell line. (**B**) Expression profile of sex-related genes affected by *h1.1-like* RNAi in the testicular cell line. (**C**) Expression profile of sex-related genes affected by *h1.2-like* RNAi in the testicular cell line. (**D**) Expression profiles of sex-related genes affected by h3 RNAi in the testicular cell line. (**E**) Expression profile of sex-related genes affected by h3.3-like RNAi in the testicular cell line. A *p*-value less than 0.05 was considered to indicate a significant difference between the two groups. In multiple comparisons, different alphabets indicated significant difference.

**Figure 7:**
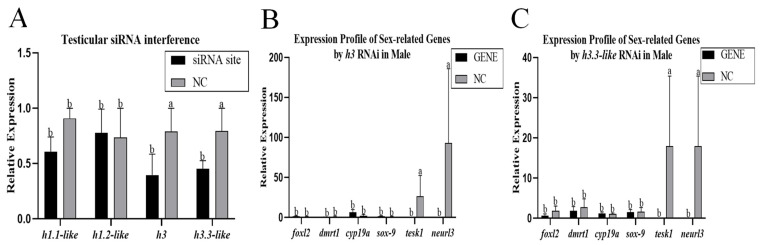
(**A**) Relative expression levels of *h1.1-like*, *h1.2-like*, *h3*, and *h3.3-like* in the testicular cell line. (**B**) Expression profile of sex-related genes affected by *h3* RNAi in the testicular cell line. (**C**) Expression profile of sex-related genes affected by h3.3-like RNAi in the testicular cell line. A *p*-value less than 0.05 was considered to indicate a significant difference between the two groups. In multiple comparisons, different alphabets indicated significant difference.

Four siRNAs were designed to knock down the testis cells of the Chinese tongue sole. Compared with those in the NC group, the expression levels of *h1.1-like*, *h1.2-like*, *h3*, and *h3.3-like* in testis cells were significantly lower after knockdown (Figure 6A). The mRNA levels of foxl2, dmrt1, cyp19a, sox-9, tesk1 and neurl3 were detected. After the *h1.1-like* site was knocked down, the expression levels of foxl2, dmrt1, cyp19a, and sox-9 significantly decreased. After the *h1.2-like* site was knocked down, the expression levels of foxl2 and cyp19a significantly increased. After the *h3* site was knocked down, the expression levels of foxl2, dmrt1, and tesk1 significantly decreased. After the *h3.3-like* site was knocked down, the expression level of dmrt1 significantly decreased. After the four sites were knocked down, the expression level of dmrt1 all significantly decreased in testis cells (Figure 6B–E). Following the role of siRNAs in Chinese tongue sole cells, siRNAs were injected into the gonads of 50-day-old male fish. Compared with those in the NC group, the expression levels of the *h3* and *h3.3-like* genes in male fish were significantly lower (Figure 7A). The mRNA levels of foxl2, dmrt1, cyp19a, sox-9, tesk1, and neurl3 were detected. After the sites of *h3* and *h3.3-like* were knocked down, the expression levels of tesk1 and neurl3 were significantly decreased in males (Figure 7B,C).

### 3.6. Results of Histone Extraction and Mass Spectrometry Data Analysis

**Figure 8:**
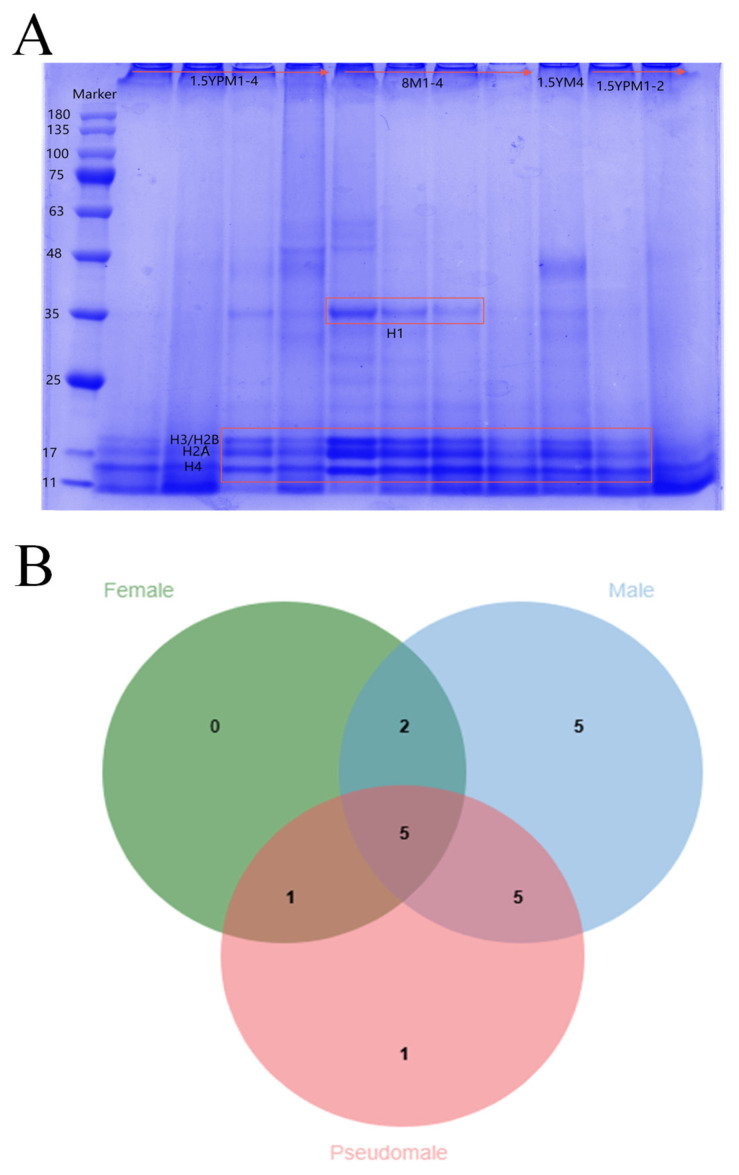
(**A**) Histone extraction experimental results. M: male; PM: pseudomale. (**B**) Venn diagram of histones of screened females, males, and pseudomales.

**Figure 9:**
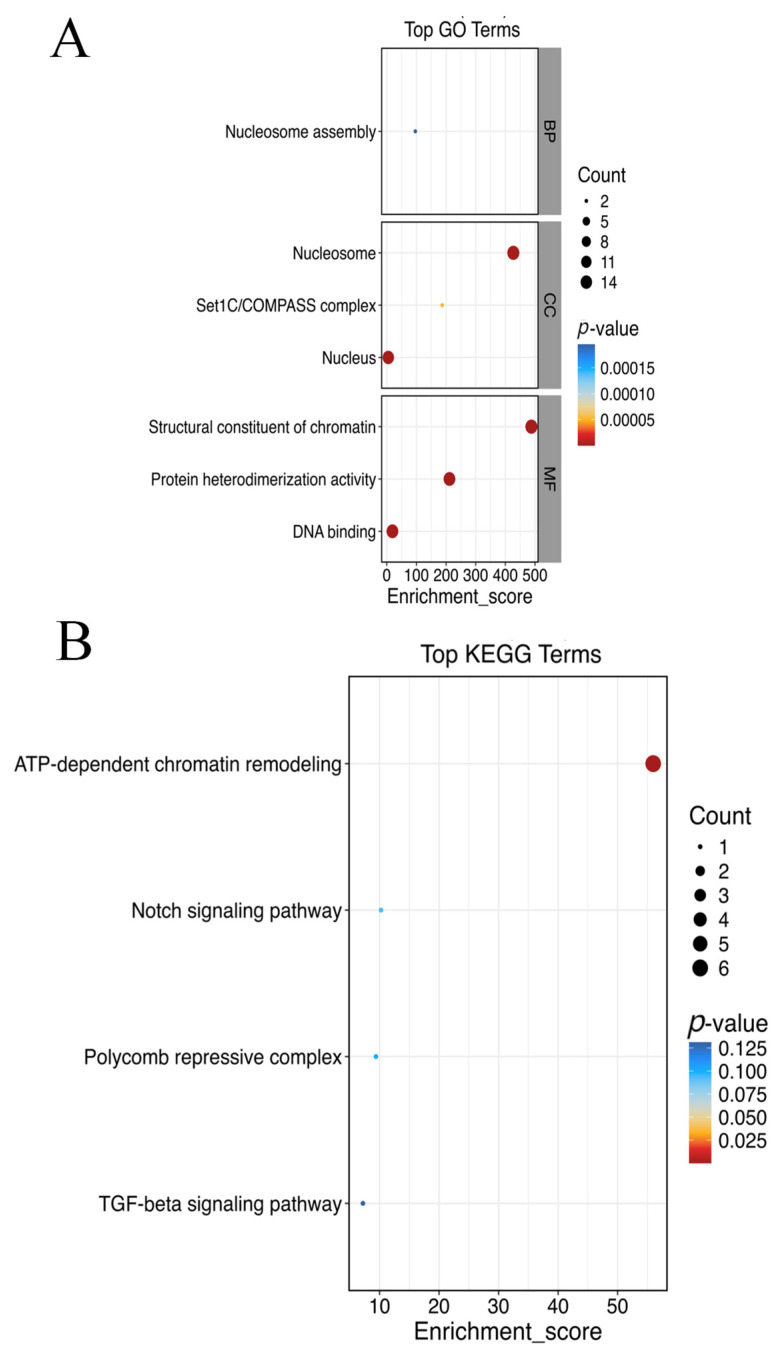
(**A**) Lollipop diagram of histones of screened females, males, and pseudomales in the GO analysis. (**B**) Lollipop diagram of histones of screened females, males, and pseudomales in the KEGG analysis.

**Figure 10:**
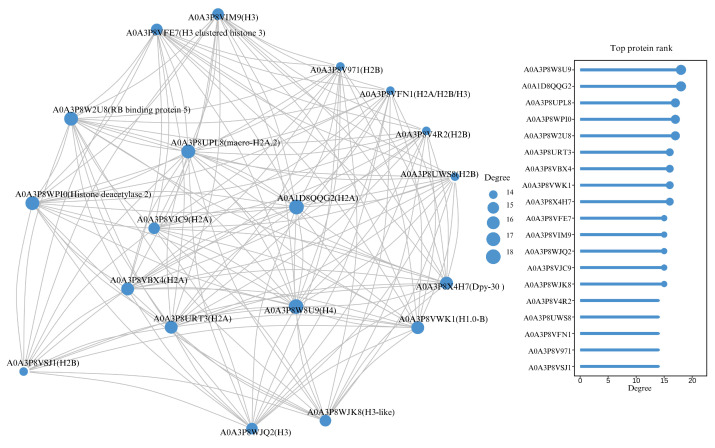
The Chinese tongue sole histones enriched in the PPI network, which discover patterns of interactions between proteins, identify key proteins, and predict protein function.

The BCA protein concentration determination kit (Solarbio, China) was used to determine the concentration of the histone mixture. The samples were run on a 12% SDS–polyacrylamide gel electrophoresis (SDS-PAGE) gel at 140 V voltage for 60 min and stained with Coomassie Blue dye. The results revealed that H1 is likely 35 kDa, H3/H2B, H2A, and H4 is likely 11–25 kDa (Figure 8A). Histone data were screened from the mass spectrometry results for comparative analysis and research (Figure 8B). Overall, the degree of histone modification in male fish is greater than that in female fish and pseudomale fish. There are more histones identified and more sites and forms of modification (Table 3). GO analysis revealed that the histones of Chinese tongue sole were involved in biological processes, cellular components, and molecular functions, with the largest proportion being structural components of nucleosomes and chromatin (Figure 9A). KEGG analysis revealed that histones were most enriched in the ATP-dependent chromosome remodeling pathway, indicating that histone genes may have a role in the chromosome remodeling pathway (Figure 9B). According to the connectivity in the PPI network, the first two histones that were enriched were H4 and H2A. Histone H2A variants have a unique role in the process of sperm chromatin packaging. Histone H4 is one of the slowest-evolving histones (Figure 10).

## 4. Discussion

Mass spectrometry analysis revealed that histones have an important role in chromosome remodeling. As an important regulatory means of spermatogenesis, chromatin remodeling mainly occurs through the post-translational modification (PTM) of integrating testis-specific histone variants [22,23]. H1.1~H1.5 are the main types of H1 that are commonly expressed in somatic cells, but their expression is strictly regulated in different tissues and cell types [24]. H1 histones are thus involved in the formation of higher-order chromatin structures, and they modulate the accessibility of regulatory proteins, chromatin remodeling factors, and histone modification enzymes to their target sites. The major posttranslational modification of H1 histones is phosphorylation, which reaches a peak during G2 and mitosis. Phosphorylation is, however, also involved in the control of DNA replication, and it contributes to the regulation of gene expression [25]. The core histone H3-H3 interface, conserved between eukaryotes and archaea, carries an ancestral copper reductase function [26]. H3.3, also called a replacement variant, is ancestral to its replicative counterparts, H3.1 and H3.2 [27]. H3.3 are non-replicative histones that do not reach the peak in the S phase, which helps form an open chromatin configuration. H3.3 is required for chromatin recombination and histone–protamine substitution [28]. The gene encoding histone H3.3 is continuously expressed throughout the cell cycle and enables histone variant H3.3 to integrate into chromatin in a DNA-independent manner, and histone variant H3.3 has an important role in transcription, genomic stability, and mitosis [29,30,31]. Interference with the H3.3 gene causes male sterility [32,33,34,35], as there are defects in the chromatin recombination of germ cells, and normal protamine incorporation also fails [36,37].

After a siRNA-mediated knockdown in the testis, the expression of multiple genes was affected. Foxl2, dmrt1, cyp19a, sox-9, tesk1, and neurl3 were upregulated or downregulated to varying degrees. The experimental results revealed that after the four sites were knocked down, the expression level of dmrt1 all significantly decreased in testis cells; after the sites of *h3* and *h3.3-like* were knocked down, the expression levels of tesk1 and neurl3 were significantly decreased in male. According to reports, the role of the Foxl2 gene and doublesex-mab3-related transcription factor 1 (Dmrtl) is particularly important in the process of male and female sex differentiation, and the regulation of these two genes has opposite effects. If the Foxl2 gene is knocked out, sex reversal will occur in goats, frogs, and some fish [38]. The Cyp19a gene encodes gonadal aromatase (CYP19a1), which is expressed mainly in fish ovaries and is slightly expressed in the testis and brain [39]. The Cyp19a gene is also sexually dimorphic and highly specifically expressed in the ovarian tissue of adult Chinese soft-shelled turtles but not in the testis tissue [40]. tesk1 mRNA is expressed mainly in the testes of the Chinese tongue sole [41]. The E3 ubiquitin ligase genes neurl3 play important roles in male gonadal differentiation and spermatogenesis during spermatogenesis in the Chinese tongue sole [42]. These data together suggest that *h3* and *h3.3-like* might play a negative role in spermatogenesis, or there may be a complementary regulation in the chromosome remodeling pathway. Z chromosome relative length is 4.88 ± 0.44, and W chromosome relative length is 2.91 ± 0.57. The W chromosome has about three-fifths of the Z chromosome in relative length [43]. Is that why W sperms are less sustainable than Z sperms? If the phenotypic effects can be observed by long-term knockdown, the network of spermatogenesis and the disappearance of W sperms may be better explained. It is interesting that the expression levels of *h1.1-like*, *h3*, and *h3.3-like* were highest at 2Y, and *h1.2-like* was highest at 1.5Y in the testis, while the role in females requires further investigation.

The *h1.1-like*, *h1.2-like*, *h3*, and *h3.3-like* promoter clones have obvious activity and multiple transcription factor-binding sites related to sex differentiation. The experimental results revealed that the promoter activity of the *h1.1-like*, *h1.2-like*, *h3*, and *h3.3-like* promoter regions decreased after binding to the four transcription factors YY1A, YY1B, C-JUN, and JUNB, whereas the promoter activity of the *h3* and *h3.3-like* promoter regions increased after binding to the AR and ETS-2 transcription factors. According to reports, C-Jun and JUNB are part of the AP-1 complex. C-Jun is expressed throughout the entire process of embryonic development and organogenesis, whereas JunB is expressed in the late embryonic stage [44]. YY1 is a widely distributed transcription factor that is involved in the inhibition and activation of multiple promoters. YY1 can directly introduce histone deacetylases and histone acetyltransferases into the promoter, thereby activating or inhibiting the promoter so that histone modifications act on YY1 [45]. AR is an androgen-induced member of the nuclear receptor superfamily of transcription factors. The transcriptional activation of AR ultimately requires RNA Pol II (RNA polymerase-II) to be recruited to the promoter of the target gene. Androgen receptor (AR) signaling has an important role in the development and progression of prostate cancer [46]. As an important transcription factor, Ets-2 regulates the migration and differentiation of embryonic trophoblast cells [47]. These data together suggest the *h1.1-like*, *h1.2-like*, *h3*, and *h3.3-like* genes have important roles in transcription, genome stability, and mitosis, and their synthesis is the key to activating chromatin remodeling.

## 5. Conclusions

We selected four histone genes, *h1.1-like*, *h1.2-like*, *h3*, and *h3.3-like*, for further analysis and research. The expression levels of the *h1.1-like*, *h3*, and *h3.3-like* genes reached their highest levels at 2 yph, and the expression level of *h1.2-like* reached its highest level at 1.5 yph, indicating that its role began during the late stage of gonadal development. Promoter activity verification revealed that the promoters of the *h1.1-like*, *h1.2-like*, *h3*, and *h3.3-like* genes were located approximately upstream 2000 bp and six histone-related transcription factor sites were predicted. YY1A, YY1B, C-JUN, and JUNB may have negative regulatory effects on *h1.1-like*, *h1.2-like*, *h3*, and *h3.3-like*; AR and ETS-2 may have positive regulatory effects on *h3* and *h3.3-like*. The ISH results revealed that *h1.1-like*, *h1.2-like*, *h3*, and *h3.3-like* mRNAs were located mainly in the sperm cells in the testes and the oocytes at various stages in the ovaries. After siRNA knockdown, the expression of dmrt1 in testis cell lines and the expression of tesk1 and neurl3 in males was downregulated, suggesting that the *h1.1-like*, *h1.2-like*, *h3*, and *h3.3-like* genes may have a negative regulatory role in spermatogenesis. The regulatory role in female fish remains to be explored. Mass spectrometry analysis revealed that histones have an important role in chromosome remodeling. These results provide a genetic basis for the molecular mechanism of gonadal development and spermatogenesis in Chinese tongue sole.

## Figures and Tables

**Table 1:** The partial species in this study.

Version	Species Name
XP_016897158.1	*Cynoglossus semilaevis*
XP_014324626.1	*Xiphophorus maculatus*
XP_054866287.1	*Amphiprion ocellaris*
XP_041839043.1	*Melanotaenia boesemani*
XP_043954260.1	*Gambusia affinis*
XP_060905642.1	*Labrus mixtus*
XP_038161099.1	*Cyprinodon Tularosa*
XP_008420766.1	*Poecilia reticulata*
XP_026184457.1	*Mastacembelus armatus*
XP_051801305.1	*Acanthochromis polyacanthus*
XP_017288577.1	*Kryptolebias marmoratus*
XP_047184325.1	*Scophthalmus maximus*
XP_038550903.1	*Micropterus salmoides*
XP_047241573.1	*Girardinichthys multiradiatus*
XP_062289075.1	*Scomber scombrus*
XP_035984755.1	*Fundulus heteroclitus*
XP_060756761.1	*Neoarius graeffei*
XP_047463622.1	*Mugil cephalus*
XP_059810925.1	*Hypanus sabinus*
XP_035695241.1	*Branchiostoma floridae*
KAI3374029.1	*Scortum barcoo*
XP_026072634.1	*Carassius auratus*

**Table 2:** Bioinformatics of h1.1-like, h1.2-like, h3, and h3.3-like genes in Chinese tongue sole.

Gene	*h1.1-like*	*h1.2-like*	*h3*	*h3.3-like*
Gene ID	103395672	103379258	103379247	103394976
Chromosome	19	5	5	19
Exon number	1	1	2	5
Sequence length (bp)	1813	1136	485	743
ORF length (bp)	666 (881–1546)	621 (517–1137 )	411 (38–448 )	411 (173–583 )
Molecular formula	C_991_H_1771_N_305_O_272_S_3_	——	C_674_H_1138_N_216_O_187_S_4_	C_672_H_1130_N_214_O_188_S_3_
Amino acid number	221	207	136	136
Atom number	3342	——	2219	2207
Molecular weight (Da)	22,408.02	21,194.93	15,388.03	15,311.87
Theoretical isoelectric point (pI)	10.96	10.80	11.27	11.14
Instability coefficient	36.15	42.81	47.19	42.24
Aliphatic index	62.26	65.89	80.51	79.78
Average hydrophilicity (GRAVY)	−0.753	−0.661	−0.604	−0.638
Extinction coefficient	0	1490	4470	4470
Mature protein	8 (Asp + Glu)	9 (Asp + Glu)	11 (Asp + Glu)	11 (Asp + Glu)
	68 (Arg + Lys)	62 (Arg + Lys)	31 (Arg + Lys)	30 (Arg + Lys)

**Table 3:** Screened histones in female, male, and pseudomale Chinese tongue sole.

Sex	Accession	Description	Modifications
Male	A0A3P8VSJ1	Histone H2B	NO Modifications
	A0A3P8WPI0	Histone deacetylase 2	
	A0A3P8VJC9	Histone H2A	
	A0A3P8W2U8	RB binding protein 5, histone lysine methyltransferase complex subunit	
	A0A3P8X4H7	Dpy-30 histone methyltransferase complex regulatory subunit	
Pseudomale	A0A3P8WJK8	Histone H3-like centromeric protein A	
Female|Male	A0A3P8URT3	Histone H2A	Male: Methyl [K96(98.8)]
	A0A3P8VIM9	Histone H3	Female: Methyl [K80(100)]; Acetyl [K28(100)] Male: Methyl [K28(100); K37(99.1); K38(100); K80(100)]; Acetyl [K10(100); K15(100); K19(100); K24(100); K28(100); K80(100)]
Female|Pseudomale	A0A3P8UWS8	Histone H2B	
Male|Pseudomale	A0A3P8WJQ2	Histone H3	Male: Methyl [R53(100); K57(100); K80(100)]; Acetyl [K10(100); K15(100); K19(100); K24(100); K28(100); K80(100)] Pseudomale: Methyl [K80(100)]; Acetyl [K19(100); K24(100); K80(100)]
	A0A3P8VBX4	Histone H2A	Male: Methyl [K96(98.8)]
	A0A3P8VFN1	Histone H2A/H2B/H3 domain-containing protein	NO Modifications
	A0A3P8V971	Histone H2B	
	A0A3P8VWK1	Histone H1.0-B	
Female|Male|Pseudomale	A0A3P8W8U9	Histone H4	Female: Methyl [R14(100)]; Acetyl [K11(100)] Male: Methyl [R14(100)]; Acetyl [K11(100); K50(100)]; GG [K22(99.1)] Pseudomale: Methyl [R14(100)]; Acetyl [K11(100)]
	A0A3P8VFE7	H3 clustered histone 3	Female: Methyl [K66(100)] Male: Methyl [K66(100); R103(100)]; Acetyl [K66(100); K102(100)] Pseudomale: Methyl [K66(100)]; Acetyl [K66(100)]
	A0A3P8V4R2	Histone H2B	Male: Methyl [K54(100)]
	A0A1D8QQG2	Histone H2A	NO Modifications
	A0A3P8UPL8	Core histone macro-H2A.2	

## Data Availability

The original contributions presented in this study are included in the article/Supplementary Material. Further inquiries can be directed at the corresponding author(s).

## Supplementary Materials

The following supporting information can be downloaded at: https://www.mdpi.com/article/10.3390/ani15040593/s1, Figure S1: Hydrophilicity and hydrophobicity analysis of four histones in Chinese tongue sole; Figure S2: Analysis of four histone transmembrane regions (A) and potential signal peptide cleavage sites (B) in Chinese tongue sole; Figure S3: Analysis of four histone glycosylation sites (A) and phosphorylation sites (B) in Chinese tongue sole; Figure S4: Analysis of four histone domains in Chinese tongue sole (horizontal gray bars indicate amino acid sequences with no predictive function); Figure S5: (A–I) Localization of H1.1-like mRNA in the gonads of Chinese tongue sole. (A,B,D,E,G,H) Sense probes combined display no color. (C,F,I) Antisense probes; Figure S6: (A–I) Localization of H1.2-like mRNA in the gonads of Chinese tongue sole. (A,B,D,E,G,H) Sense probes combined display no color. (C,F,I) Antisense probes; Figure S7: (A–I) Localization of H3 mRNA in the gonads of Chinese tongue sole. (A,B,D,E,G,H) Sense probes combined display no color. (C,F,I) Antisense probes; Figure S8: (A–I) Localization of H3.3-like mRNAs in the gonads of Chinese tongue sole. (A,B,D,E,G,H) Sense probes combined display no color. (C,F,I) Antisense probes; Table S1: Primers or siRNAs used in the present study.
