# Supplementary material for: Functional Study of Four Histone Genes Involved in the Spermatogenesis of Cynoglossus semilaevis
Source: Animals (Basel). 2025 Feb 18;15(4):593. doi: 10.3390/ani15040593 (PMC11851421; doi:10.3390/ani15040593)
Supplement: Supplementary file 1 [file animals-15-00593-s001.zip › Table S1.pdf]

Table S1

Primers or siRNAs used in the present study

Primer	Sequences (5'→3')	Purpose	Product Size
h1.1-like-F	TGAACGAACACCTGACGGAAA	PCR	745 bp
h1.1-like-R	AAAAGAGCCGTTGTTTGACAGC		
h1.2-like-F	GCTGCGGGAAACTTCACAAC	PCR	674 bp
h1.2-like-R	CTTCTTGGGTGCTGCCTTCT		
h3-F	TCGCTCTAGTGAAACAGTCACA	PCR	469 bp
h3-R	GTGTTGTCAGGCTGTAGGGG		
h3.3-like-F	TACAAAGCAGACCGCCCG	PCR	454 bp
h3.3-like-R	GGTTGGTGTCCTCAAAAAGA		
Sex-F	CCTAAATGATGGATGTAGATTCTGTC	Sex identification	
Sex-R	GATCCAGAGAAAATAAACCCAGG		
h1.1-like-qF	GCTGAGTCCAAAGAGCGGAA	qPCR	153 bp
h1.1-like-qR	GATGCCTTTGGTCTGGGTCA		
h1.2-like-qF	CACTCGTGTCAAGATGTCAATCA	qPCR	182 bp
h1.2-like-qR	CTTCACGGGGGCCTTCTTTG		
h3-qF	CCCGTACTAAACAAACCGCC	qPCR	186 bp
h3-qR	GGATGAGAAGCTCCGTGGAT		
h3.3-like-qF	AAGCTCCTCGCAAACAGTTG	qPCR	182 bp
h3.3-like-qR	GATCTCCCTCACCAGACGCT		
β-actin-qF	CCTTGGTATGGAGTCCTGTGGC	qPCR	150 bp
β-actin-qR	TCCTTCTGCATCCTGTCGGC		
Sox-9-qF	AAGAACCACACAGATCAAGACAGA	qPCR	150 bp
Sox-9-qR	TAGTCATACTGTGCTCTGGTGATG		
cyp19a-qF	GGTGAGGATGTGACCCAGTGT	qPCR	230 bp
cyp19a-qR	ACGGGCTGAAATCGCAAG		
neurl3-qF	CTGGTGTTTAGCAGCCGTCCT	qPCR	234 bp
neurl3-qR	CCAGAACTCCAGCACTGACCC		
foxl2-qF	GAGAGGAAGGGCAACTACTGGA	qPCR	248 bp
foxl2-qR	TGGTTGGAAGTGCGTGGG		
dmrt1-qF	GGAGGAAGAACTTGGGATTTG	qPCR	213 bp
dmrt1-qR	AGGTAGGAGGTTGCTGGG		
tesk1-qF	GCAGAAACTCTCTCACCCCAACA	qPCR	229 bp
tesk1-qR	CCAGACCAAAGTCCGTCACCA		
h1.1-like-dF	CGAGATCTGCGATCTAAGTAAAGCTTACGGGGA GGATTTGTTTT	promoter cloning	1928 bp
h1.1-like-dR	AACAGTACCGGAATGCCAAAGCTTCTGTTGTAG TTGTGCTCAT		
h1.2-like-dF	CGAGATCTGCGATCTAAGTAAAGCTTTCACCGT CATCTGTGT	promoter cloning	1675 bp
h1.2-like-dR	AACAGTACCGGAATGCCAAAGCTTTCACGTAAC GGTGGAG		

h3-dF	CGAGATCTGCGATCTAAGTAAAGCTTTTGCCGAGACCCT	promoter cloning	1940 bp
h3-dR	AACAGTACCGGAATGCCAAAGCTTAGGAAAAGAAGGCGCT		
h3.3-like-dF	CGAGATCTGCGATCTAAGTAAAGCTTTTCCCACATTTGCTACCC	promoter cloning	1974 bp
h3.3-like-dR	AACAGTACCGGAATGCCAAAGCTTTTGCCTCCCTTTGTTG		
h1.1-like-tF	ATTTAGGTGACACTATAGAACCAAGAAGGAGCCGACGG	ISH	448 bp
h1.1-like-tR	TAATACGACTCACTATAGGGTTCTTGCTCTTCTTGGCTGCAG		
h1.2-like-tF	ATTTAGGTGACACTATAGAAGCGCCAAAGGATACGATGTG	ISH	441 bp
h1.2-like-tR	TAATACGACTCACTATAGGGCTTCTTGGGTGCTGCCTTCT		
h3-tF	ATTTAGGTGACACTATAGAATACCGGCGGTGTGAAGAAG	ISH	352 bp
h3-tR	TAATACGACTCACTATAGGGTTAAGCCCTCTCTCCGCG		
h3.3-like-tF	ATTTAGGTGACACTATAGAAATGGCTCGTACAAAGCAGACC	ISH	354 bp
h3.3-like-tR	TAATACGACTCACTATAGGGTGGTGACACGCTTAGCAT		
h1.1-like-464-F	AAGAAAACUGCGGCGAAGAAATT	siRNA site	
h1.1-like-464-R	UUUCUUCGCCGCAGUUUUCUUTT		
h1.2-like-264-F	CGGCUCUUUCAAGAUGAACAATT	siRNA site	
h1.2-like-264-R	UUGUUCAUCUUGAAAGAGCCGTT		
h3-331-F	CACCAUCAUGCCCAAAGACAUTT	siRNA site	
h3-331-R	AUGUCUUUGGGCAUGAUGGUGTT		
h3.3-like-346-F	CACCAUCAUGCCCAAAGACAUTT	siRNA site	
h3.3-like-346-R	AUGUCUUUGGGCAUGAUGGUGTT		
NC-F	UUCUCCGAACGUGUCACGUTT	negative control	
NC-R	ACGUGACACGUUCGGAGAATT		
