# Supplementary figures and images for: Functional Study of Four Histone Genes Involved in the Spermatogenesis of Cynoglossus semilaevis
Source: Animals (Basel). 2025 Feb 18;15(4):593. doi: 10.3390/ani15040593 (PMC11851421; doi:10.3390/ani15040593)

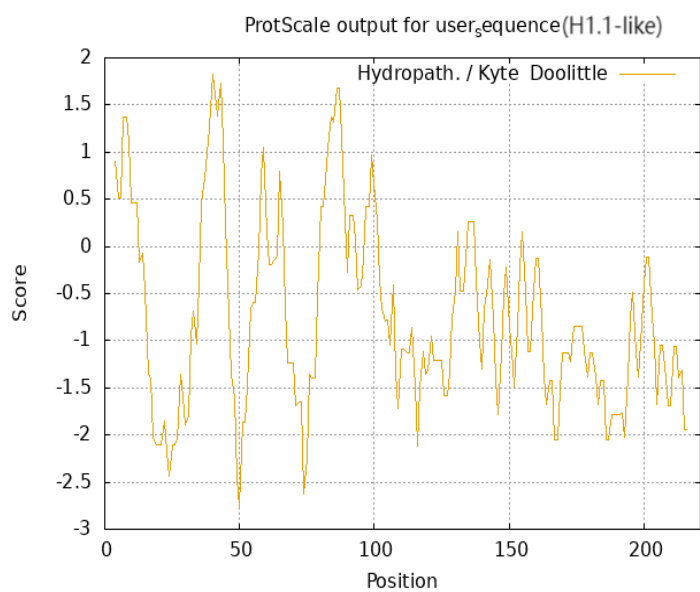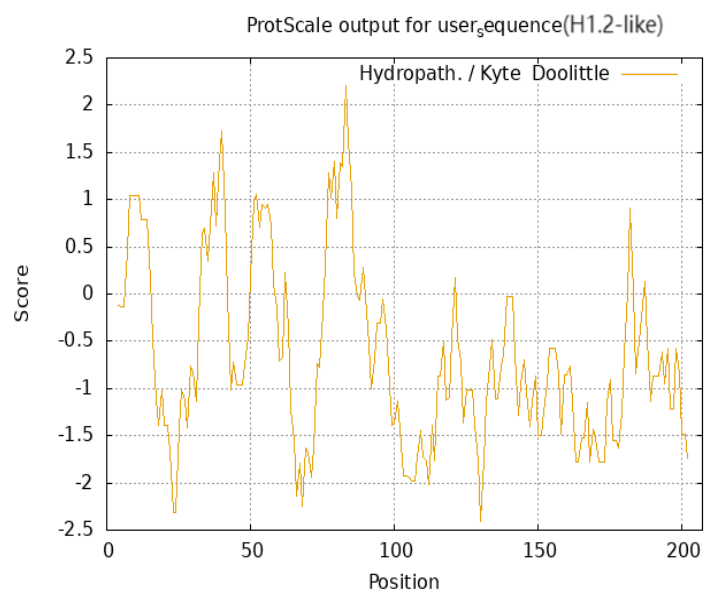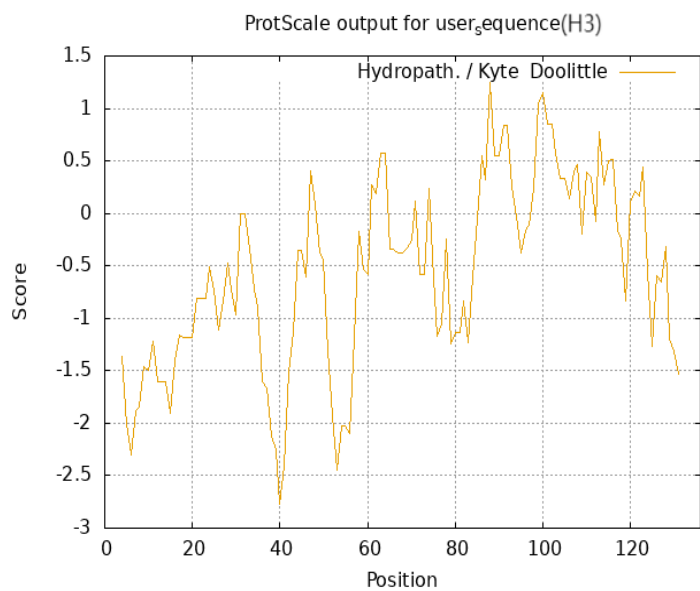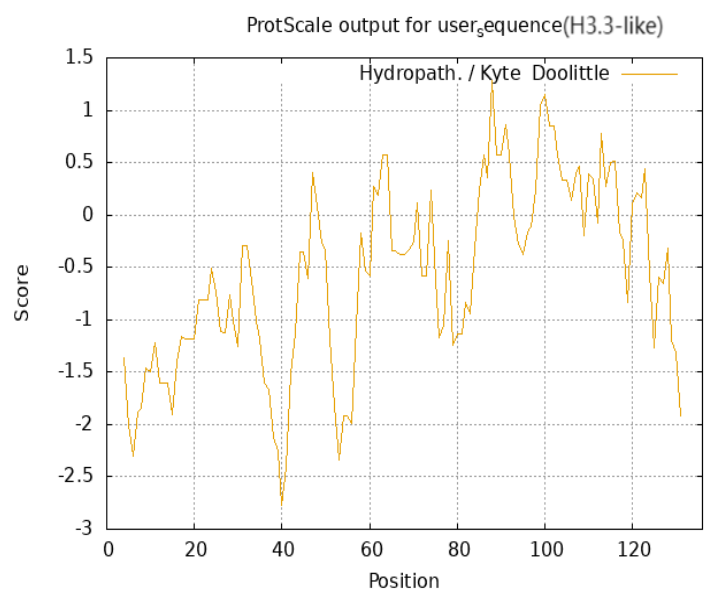

Supplement: Supplementary file 1 [file animals-15-00593-s001.zip › Figure S1.pdf]

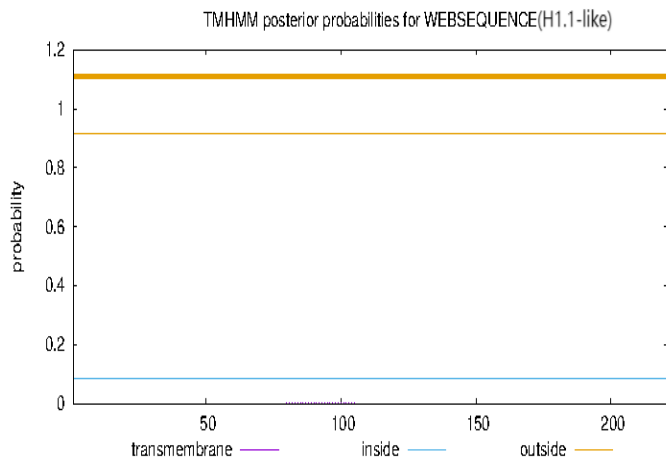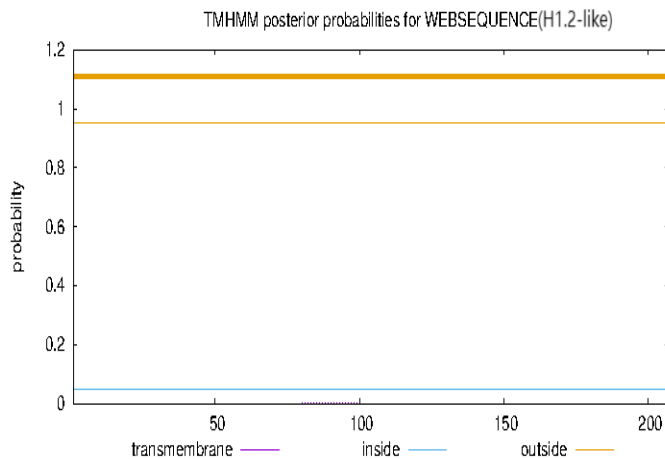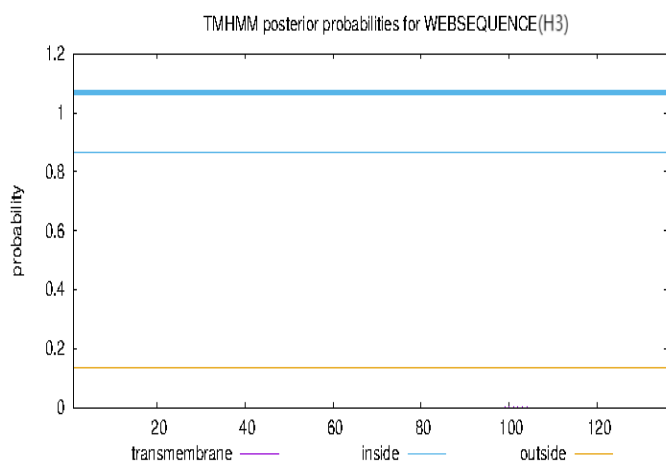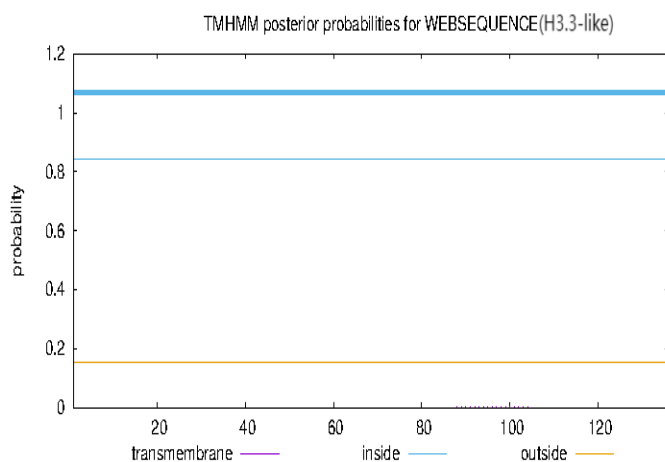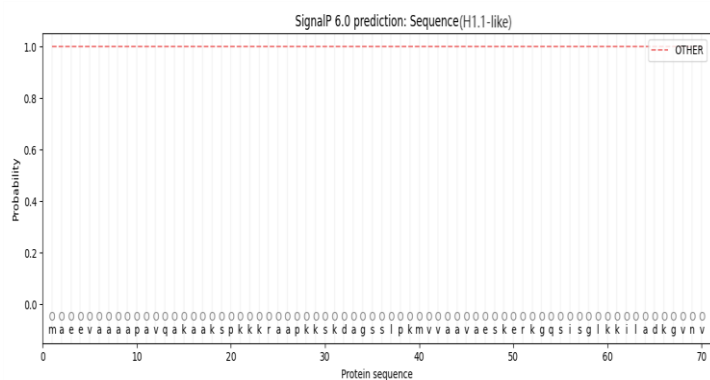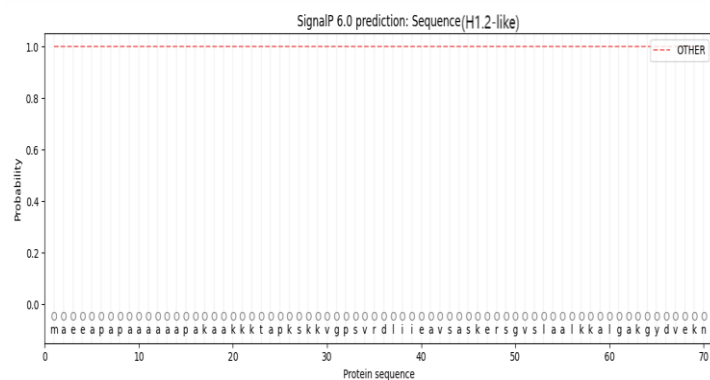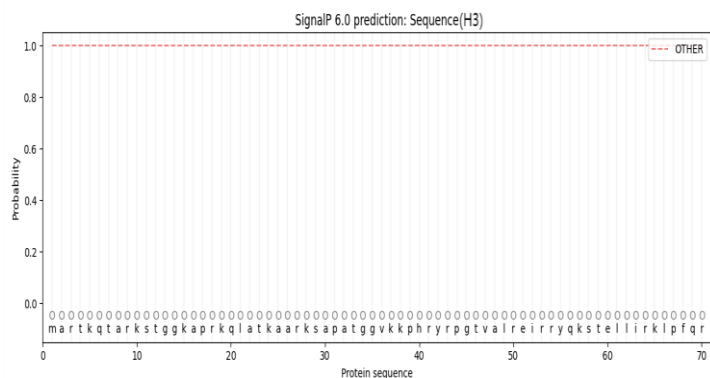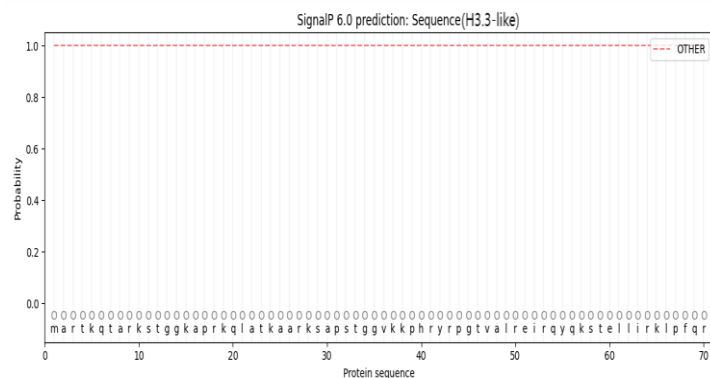

Supplement: Supplementary file 1 [file animals-15-00593-s001.zip › Figure S2.pdf]

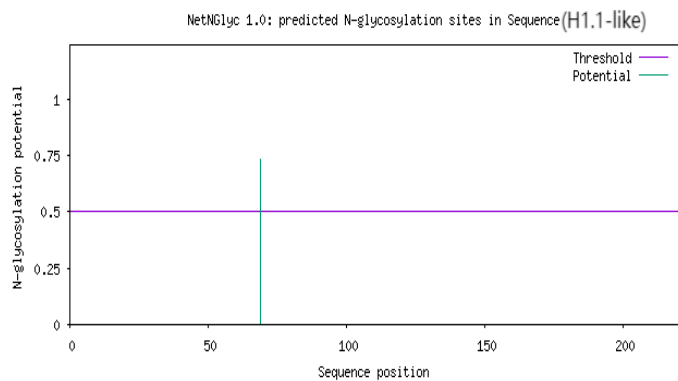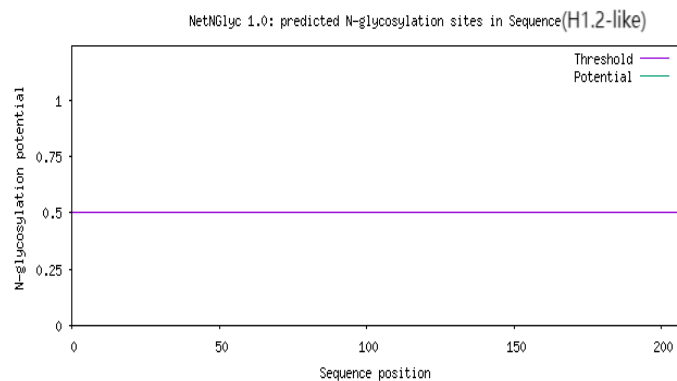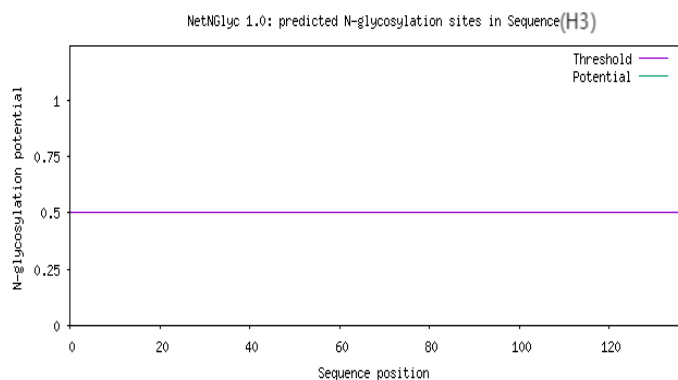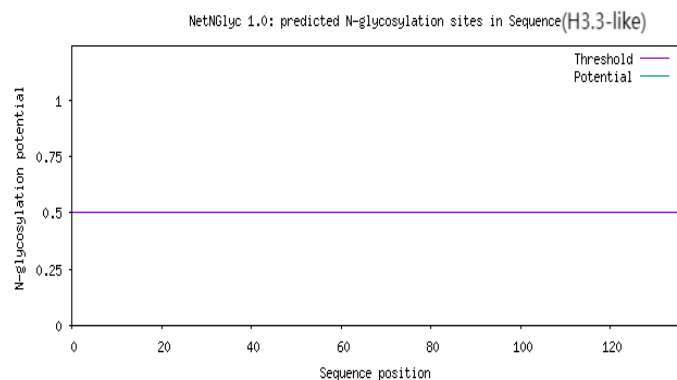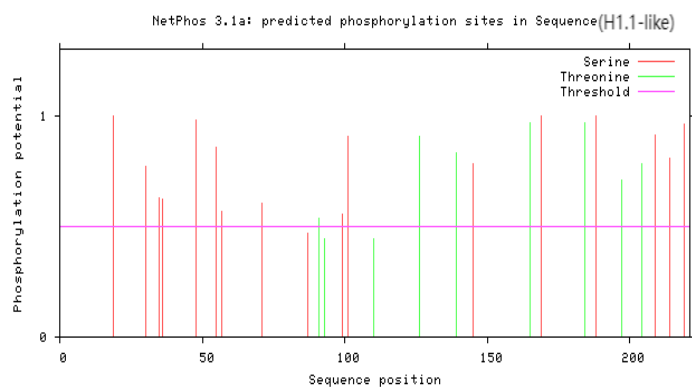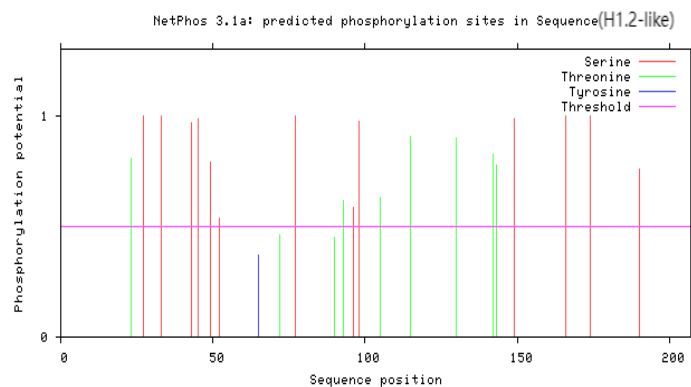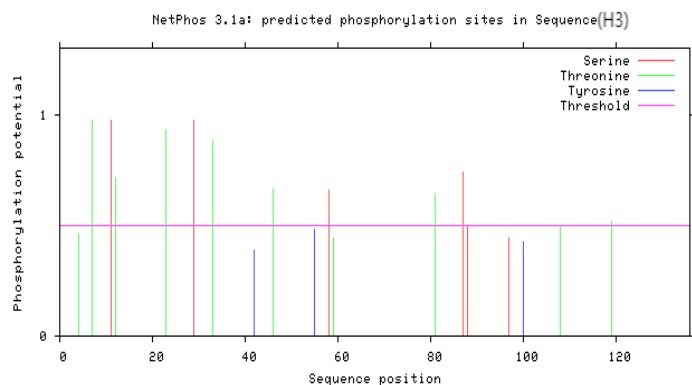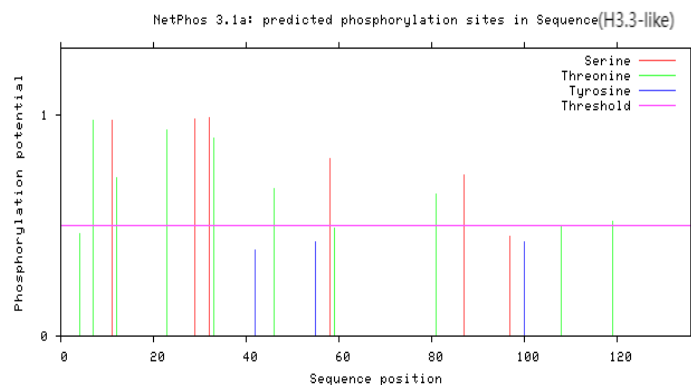

Supplement: Supplementary file 1 [file animals-15-00593-s001.zip › Figure S3.pdf]

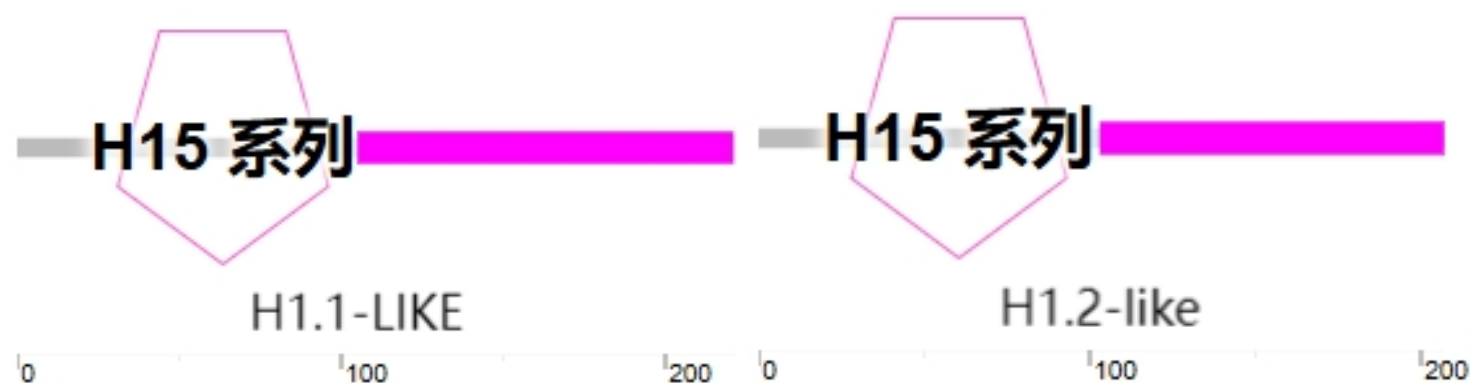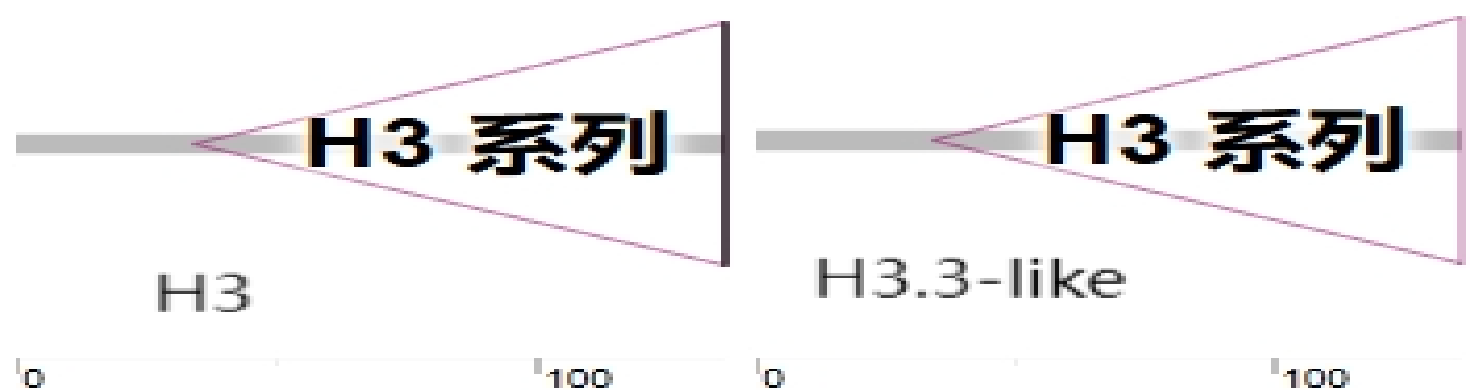

Supplement: Supplementary file 1 [file animals-15-00593-s001.zip › Figure S4.pdf]

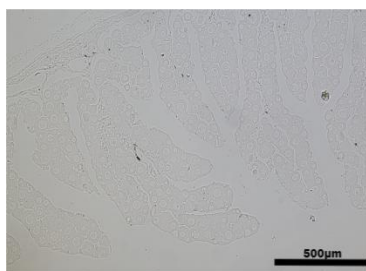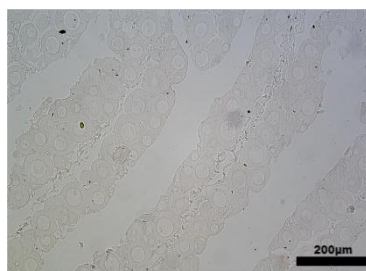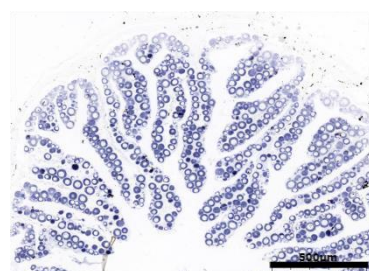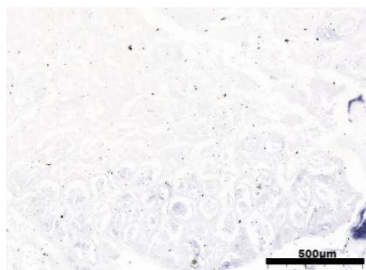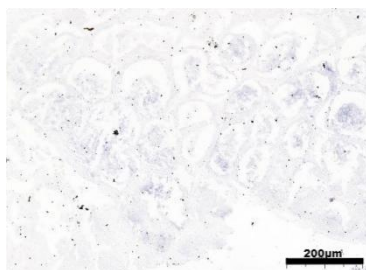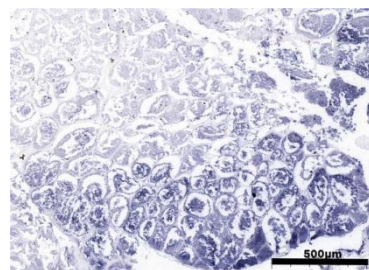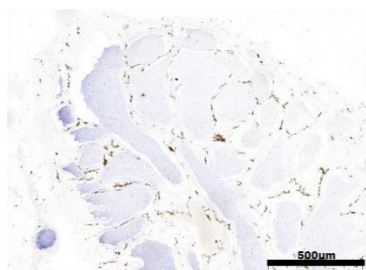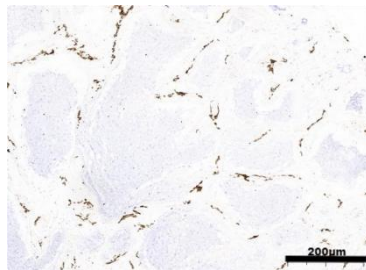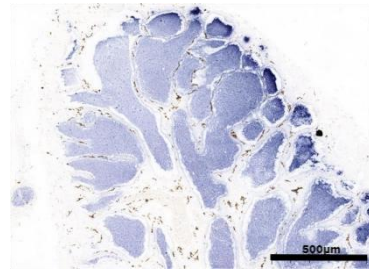

Supplement: Supplementary file 1 [file animals-15-00593-s001.zip › Figure S5.pdf]

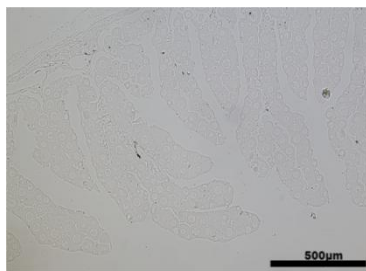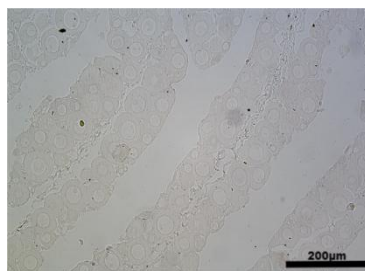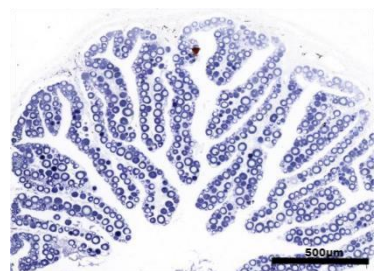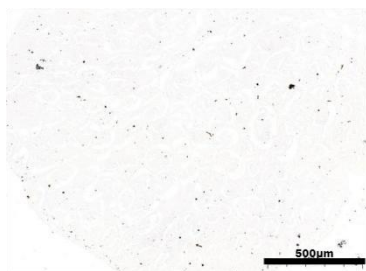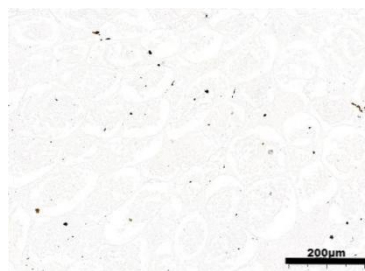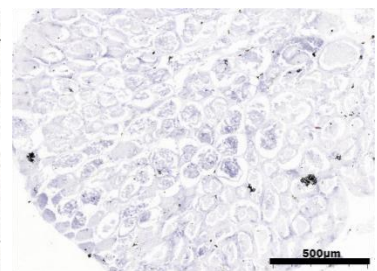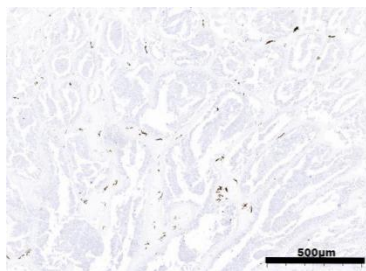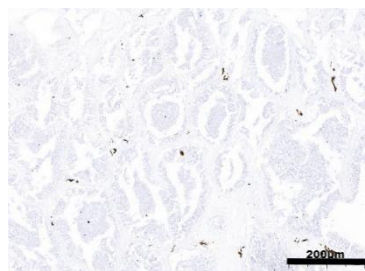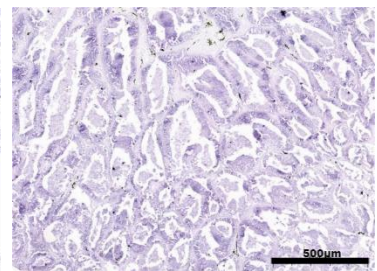

Supplement: Supplementary file 1 [file animals-15-00593-s001.zip › Figure S6.pdf]

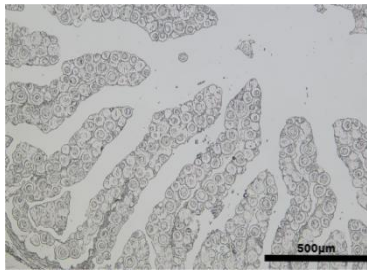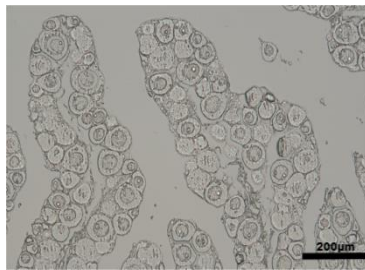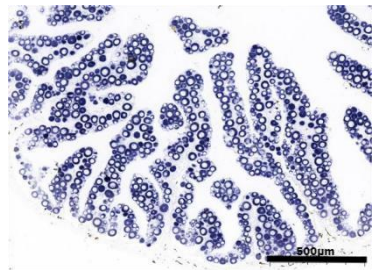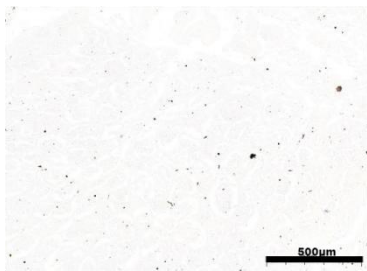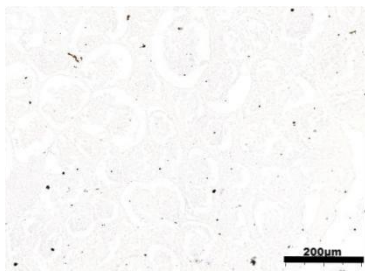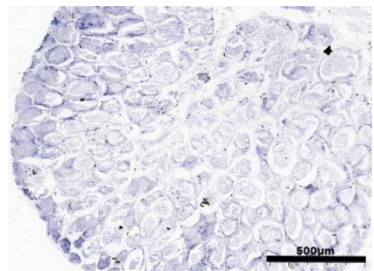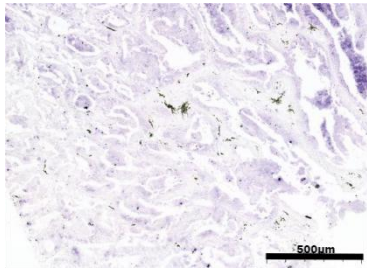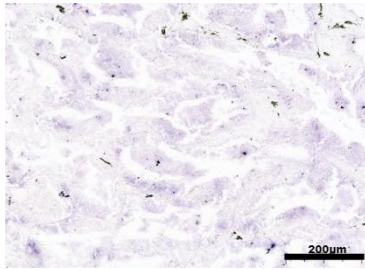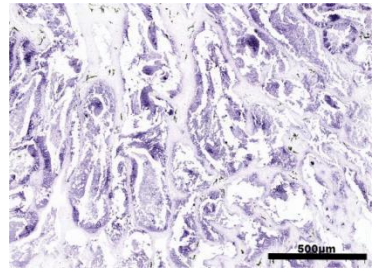

Supplement: Supplementary file 1 [file animals-15-00593-s001.zip › Figure S7.pdf]

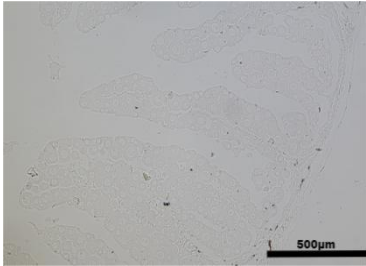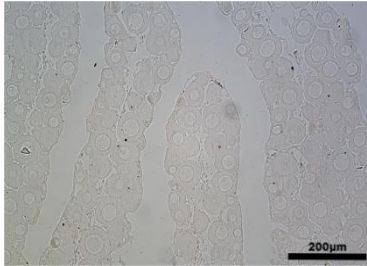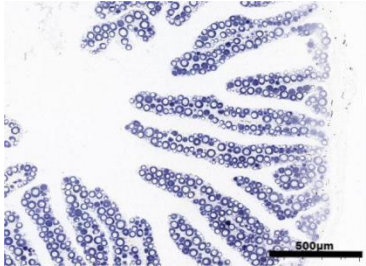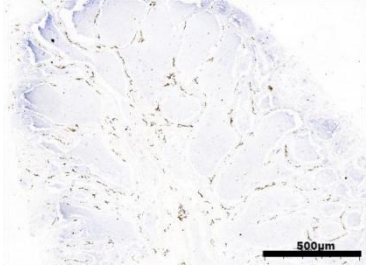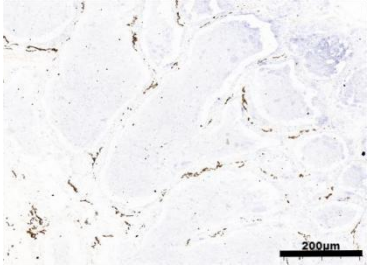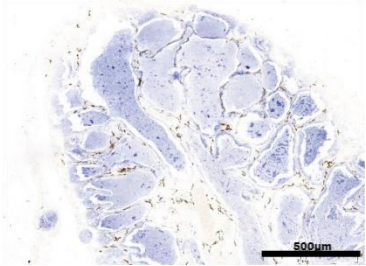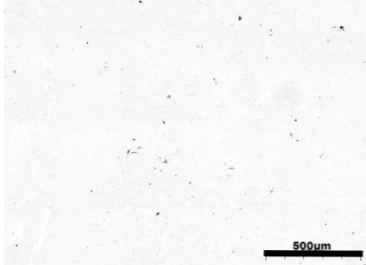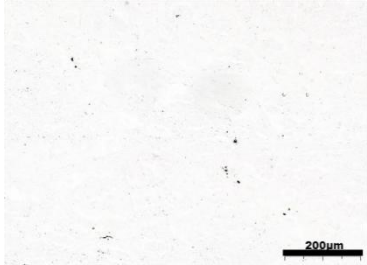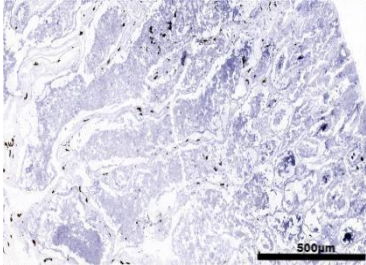

Supplement: Supplementary file 1 [file animals-15-00593-s001.zip › Figure S8.pdf]
